# Integration of transcriptomics, proteomics, and metabolomics data for the detection of the human pathogenic *Prototheca wickerhamii* from a One Health perspective

**DOI:** 10.3389/fcimb.2023.1152198

**Published:** 2023-05-05

**Authors:** Jian Guo, Juan Chen, Teng Li, Lei Gao, Cizhong Jiang, Wenjuan Wu

**Affiliations:** ^1^ Department of Laboratory Medicine, Shanghai East Hospital, School of Life Sciences and Technology, Tongji University, Shanghai, China; ^2^ Key Laboratory of Spine and Spinal Cord Injury Repair and Regeneration of Ministry of Education, Orthopaedic Department of Tongji Hospital, Shanghai Key Laboratory of Signaling and Disease Research, Frontier Science Center for Stem Cell Research, School of Life Sciences and Technology, Tongji University, Shanghai, China; ^3^ Microscopy Core Facility, Biomedical Research Core Facilities, Westlake University, Hangzhou, Zhejiang, China

**Keywords:** prototheca, algae, protothecosis, metabolomics, transcriptomics

## Abstract

*Prototheca* species are the only microalgae known to cause opportunistic infections in vertebrates and humans. Most cases of protothecosis in humans are caused by *Prototheca wickerhamii*, but knowledge of the pathogenicity and biology of *Prototheca* is limited. Globally, the diagnostic rate of *Prototheca* species infection is much lower than the actual rate of *P. wickerhamii*. The precise mechanisms underlying the pathogenesis of *Prototheca* infections remain unclear. In this study, we identified a strain of *P. wickerhamii* with atypical colony morphology. To reveal the morphological differences between *P. wickerhamii* S1 (mucous) and the molecular basis of its pathogenicity, the transcriptomics, proteomics, and metabolomics of two pathogenic *P. wickerhamii* strains and one environmental strain were analysed. Interestingly, mannan endo-1,4-β-mannosidase was significantly downregulated in *P. wickerhamii* S1, which contributes to a thinner cell wall in S1 compared to strains with typical colony morphology, and the toxicity of macrophages is reduced. Metabolite analysis revealed that the mucoid appearance of *P. wickerhamii* S1 may have been caused by an increase in linoleic acid, glycerol, and other metabolites. There is still a need to better understand the ecology, aetiology, and pathogenesis of *P. wickerhamii*, and in particular, its transmission between humans, animals, and the environment, from a One Health perspective.

## 1 Introduction

Wilhelm Krüger, a German plant physiologist, first discovered the green algae *Prototheca* in 1894 (Krüger, 1894). The first case of *Prototheca* infection in humans was described in a 31-year-old farmer in 1961 (Nelson et al., 1987). Considering that the colony morphology was similar to that of yeast-like fungi, *Prototheca* was initially identified as a fungus. Potential virulence factors involved in protothecosis have been identified using genomics, and many *P. wickerhamii* pathogenicity genes have been associated with fungi (Bakuła et al., 2021). However, the genus *Prototheca* has recently been classified into the family of the green algae Chlorellaceae. Of the 18 *Prototheca* species, only *P. wickerhamii*, *P. blaschkeae*, *P. bovis*, *P. cutis*, *P. miyajii*, and *P. ciferrii* have been implicated as vertebrate and human pathogens (Kano et al., 2022). *P. bovis* is the most virulent and causes most bovine mastitis cases. *P. wickerhamii* has rarely been described in milk from cows with mastitis; however, recently, *P. wickerhamii* was identified as a pathogenic *Prototheca* causative agent of bovine mastitis (Marques et al., 2006). *P. wickerhamii* can infect and survive in macrophages, resulting in persistent infection. The predominant *Prototheca* microalga isolated from cat protothecosis is *P. wickerhamii* (Masuda et al., 2021). In a three-year-old goat, protothecosis was caused by *P. wickerhamii* with a slowly progressive infection causing necrotising lesions and multifocal pyogranulomatous lesions on the skin of the face and head (Camboim et al., 2011). *P. wickerhamii* can cause underweight carp (Jagielski et al., 2017), and strains can be isolated from pigeons drinking wastewater (Rosario Medina et al., 2017). *P. wickerhamii* colonises flower bract water and fresh stumps (Pore, 1985) and was isolated, as the most abundant species, from raw sludge and sewage from wastewater treatment plants. *P. wickerhamii* can colonise the fingernails, skin, digestive system, and respiratory tract of humans. Furthermore, *Prototheca* microalgal infections may progress after contamination of wastewater and soil. (Kano, 2020).

For more knowledge of this fungal-like pathogen, the Medical Phycology (protothecosis and chlorellosis) Symposium was held during the 20^th^ Congress of the International Society for Human and Animal Mycology (Masuda et al., 2021). The clinical forms of human protothecosis include cutaneous lesions, olecranon bursitis, and disseminated and systemic infections. More than 50% of the cases were skin infections, and other miscellaneous cases included wounds, nail lesions, olecranon bursitis, peritonitis, blood poisoning, and disseminated infections. With an improvement or cure rate of approximately 78%, skin infections have a first-class prognosis. Blood and disseminated infection cases have the worst prognosis, with a mortality rate of approximately 56% and 33% improvement or cure (Todd et al., 2018). Surgery was the most common cause of hospital-acquired infections. When skin injuries occur in patients colonised with predisposing factors, they may become infected after exposure to water contaminated with *P. wickerhamii*. Since the first human outbreak of *Prototheca* in 2018, it has become necessary to understand the ecology and pathogenesis in humans, environments, and animals from a One Health perspective (Libisch et al., 2022). As an increasing number of cases are reported worldwide, the impact of *Prototheca* infection on human and animal health is increasing. *Prototheca* has been isolated from natural water sources and artificial aquatic reservoirs (Jagielski et al., 2022). The primary source of *Prototheca* isolates is wastewater from cities, with *P. wickerhamii* being the most common isolate (Pore et al., 1983). In Poland, a study on the occurrence of *Prototheca* in freshwater ecosystems identified *P. wickerhamii* (17/51, 33%) as the most abundant *Prototheca* microalga, followed by *P. pringsheimii*, *P. cerasi*, *P. bovis*, *P. ciferrii*, *P. cookei*, and *P. zopfii*. *P. wickerhamii* is the major isolate collected from rivers and streams in the environment (Jagielski et al., 2022).

*Prototheca* species have a two-layered cell wall (Nelson et al., 1987). The *Prototheca* cell wall is different from fungal and plant cell walls and does not contain chitin or cellulose. *Prototheca* has a thin outer layer and a thick inner layer. The release of *P. wickerhamii* endospores occurs every 5–6 h with adequate nutrients (Kano, 2020). The size of *P. wickerhamii* sporangia is 3–10 μm and is smaller than that of *P. bovis* and *P. ciferrii* (Jagielski et al., 2019a). *Prototheca* biofilms increase resistance to antimicrobial agents (Shave et al., 2021). It is necessary to apply a One Health perspective, which includes the environment, humans, and animals (Jinatham et al., 2021). To investigate the morphological differences and the molecular basis for the pathogenicity of different *P. wickerhamii* strains, we analysed transcriptomics, proteomics, and metabolomics data to determine the possible pathogenesis.

## 2 Methods

### 2.1 Sample preparation and collection

*P. wickerhamii* S1 (mucous) was isolated from an 85-year-old female patient with a skin infection in Shanghai, China (Guo et al., 2022). *P. wickerhamii* HN01 (rough) was isolated from a 45-year-old male patient in Hunan, China, who had suffered from multiple skin infections and a bloodstream infection. *P. wickerhamii* ATCC 16529 was purchased from Thermo Fisher Scientific (Waltham, MA, USA). The strains were streak-inoculated onto Sabouraud dextrose agar (SDA) (Thermo Fisher Scientific) and incubated at 35°C for 3–5 days. Then, the colonies were harvested and washed with sterilised water (Jagielski et al., 2017). Primers used for PCR amplification and sequencing of the partial CYTB gene were cytb-F: GYGTWGAACAYATTATGAGAG-5’ and cytb-R: WACCCATAARAARTACCATTCWGG. Primers were used for both amplification and sequencing of degenerate nucleotides: Y, C, or T; R, A, or G; W, A, or T. The sequence of the CYTB gene of the strains in this study was aligned with the corresponding sequences retrieved from *P. wickerhamii* from the GenBank database.

### 2.2 Phenotypic characterization of *P. wickerhamii*

Clinical strains S1 and HN01 of *P. wickerhamii* were subjected to micromorphological examination for phenotypic analysis. The morphological features of the cells were investigated on direct wet-mounted smears from SDA using an Olympus BX51 light and fluorescence microscope (Olympus Instruments Co., Tokyo, Japan). Images were taken using an Olympus U-TV0.63XC digital camera connected to a microscope. Olympus cellSens Standard 3.1 software was used for morphometric studies. *P. wickerhamii* cells were selected for fluorescence microscopy using the one-step fungal fluorescence staining method (Baso Diagnostic, Inc. Zhuhai, China).

### 2.3 Transmission electron microscopy

*P. wickerhamii* S1 and HN01 were first picking into 20% BSA in cacodylate buffer (20 μL) and 0.8 μL BSA was transferred with a few samples to a 3 mm-diameter,100 μm-deep well of an aluminium carrier disk (Engineering Office of M. Wohlwend), quickly capped with a 0.16 mm × 3 mm sapphire disc (flat side coated with hexadecene), loaded into a customised 0.66 mm × 3.05 mm HPF specimen holder for rapid freezing at ~2,050 bar pressure for 340–360 ms using a Leica ICE HPF machine, and kept in liquid nitrogen until use.

The specimens were treated with a cocktail of 1% osmium tetroxide, 0.2% uranyl acetate, 3% H_2_O in acetone, and 5% methanol at -90°C for 72 h. The temperature was raised to -60°C for 15 h and kept at -60°C for 24 h, then raised to -20°C for 15 h, and increased to 20°C from 2 to 17 h. This was followed by four washes with 100% acetone, each for 15 min. Subsequently, the specimens were incubated with 1% thiocarbohydrazide in 80% methanol at room temperature (RT) for 60 min, followed by six washes, each for 10 min, with 100% acetone. The specimens were stained with 2% osmium tetroxide in acetone at RT for 1 h and washed with 100% acetone four times each for 15 min, followed by incubation in 0.5% uranyl acetate in acetone in the dark overnight at 4°C. After four washes in pure acetone, each for 15 min, the samples were incubated in Epon resin with pure acetone for 3 h (at a ratio of 1:3), 3 h (at a ratio of 1:2), 3 h (at a ratio of 1:1), 3 h (at a ratio of 3:1), and 6 h each time, with four repetitions (100% Epon resin) on a rocker. The samples were embedded with freshly made resin and cured overnight at 38°C and then polymerised in a 60°C oven for 48 h. For transmission electron microscopy (TEM), samples were sliced (approximately 70 nm thick) using a Leica EM UC7 ultramicrotome and collected on copper grids. Sections of spermatids collected by FACS were stained with 2% uranyl acetate and Sato’s triple lead. All sections were imaged using a Talos L120C TEM (Thermo Fisher Scientific) at 80 kV, equipped with a 4 k × 4 k Ceta CCD camera.

### 2.4 Scanning electron microscopy

The liquid cultures of *P. wickerhamii* S1 and HN01 were centrifuged at 10,000 rpm. The pellets were washed with 0.1 M sodium phosphate buffer (PB) pH 7.3 three times. The pellets were then fixed with 2.5% glutaraldehyde (25% glutaraldehyde ampules; Ted Pella Co., Redding, CA, USA) and 2% paraformaldehyde (16% paraformaldehyde; Ted Pella Co.) mixture in PB by incubating at RT for 30 min, then incubating at 4°C overnight. The pellet was washed three times with PB and collected by centrifugation. The samples were dehydrated using different ethanol volumes (30, 50, 70, 80, and 90%), and for each ethanol volume, incubated on ice for 10 min. Samples were incubated in 100% ethanol at RT three times (total of 1 h). The samples were dried using a critical-point dryer with a final rinse in pure ethanol. The dried samples were mounted on stubs containing carbon adhesives and a palladium coating of approximately 6 nm. For scanning electron microscopy (SEM), the samples were placed in a Zeiss Gemini Smart FE-SEM 550 (Zeiss, Germany) and imaged at 1.5 Kv and 4 k × 4 k.

### 2.5 Transcriptome data analysis

Nine samples (three *P. wickerhamii* strains ATCC16529, S1, and HN01, each with three biological replicates) were included in this study. The RNA-seq raw reads were preprocessed using SOAPnuke v1.4.0 (Yuxin et al., 2018). The clean reads were then mapped to the reference gene sequence (Bowtie 2 v2.2.5) (Ben and Salzberg, 2012) to acquire the position information and unique read features of the nine sequenced samples. The gene expression levels of individual samples were then calculated as fragments per kilobase per million mapped reads (FPKM) using RSEM v1.2.8 (Li and Dewey, 2011). Significantly differentially expressed genes (DEGs) between the two *P. wickerhamii* strains were identified using DEseq 2 (fold change > 2 and P < 0.05) (Love et al., 2014).

### 2.6 Proteome data analysis

The MSstats R package (Meena et al., 2014) was used to quantify the peptides and proteins from the mass spectrometry data of the 12 samples (three *P. wickerhamii* strains, each with four biological replicates). The MSstats R package was also used for intrasystem error correction and normalisation for each sample. Significantly differentially expressed proteins (DEPs) between the two types of *P. wickerhamii* strains were identified with a fold change > 2 and P < 0.05. For the functional annotation of a protein set, the protein sequences were aligned to Eukaryotic Orthologous Groups (KOG) using BLASTp with an E-value of 1E−5 or less (Koonin et al., 2004).

### 2.7 Metabolome data analysis

The 18 sample result files (three *P. wickerhamii* strains, each with six biological replicates) from Compound Discoverer were input into MetaX for data preprocessing and further analysis. After the log 2 transformation of the data, a PLS-DA model was established to screen the differential metabolites between two types of *P. wickerhamii* strains, with the scaling method Pareto and a 7-fold cross-validation. Significantly differential metabolites were defined as fold change > 2, P-value < 0.05, and variable importance for projection (VIP) >1. Pathway enrichment analysis of significantly differentially expressed metabolites was performed using the Kyoto Encyclopedia of Genes and Genomes pathway database (https://www.kegg.jp/kegg/pathway.html).

### 2.8 Macrophage cytotoxicity assay

Exponentially growing cells of the three *P. wickerhamii* strains were cocultured with bone marrow-derived macrophages (BMDMs) (multiplicity of infection [MOI] = 1:1) at 37°C with 5% CO_2_ for 6 and 24 h, respectively. After incubation, the cells were fixed with 4% formaldehyde, washed, stained with calcofluor white (10 min co-incubation), and observed under a fluorescence microscope. The concentration of lactose dehydrogenase (LDH) was assayed to determine the cytotoxicity of the three *P. wickerhamii* strains (CytoTox 96 Non-Radioactive Cytotoxicity Assay; Promega, Madison, WI, USA). Released LDH in culture supernatants was measured for 30 min, coupled with an enzymatic assay, which was used to measure the membrane integrity for cell-mediated cytotoxicity assays to measure the lysis of target cells by the three *P. wickerhamii* strains.

### 2.9 Statistical analysis

Statistical tests were performed using the Student’s t-test and Wilcoxon test. Statistical significance was set at P < 0.05.

## 3 Results

### 3.1 Description of *P. wickerhamii* strains HN01 and S1

**Figure 1:**
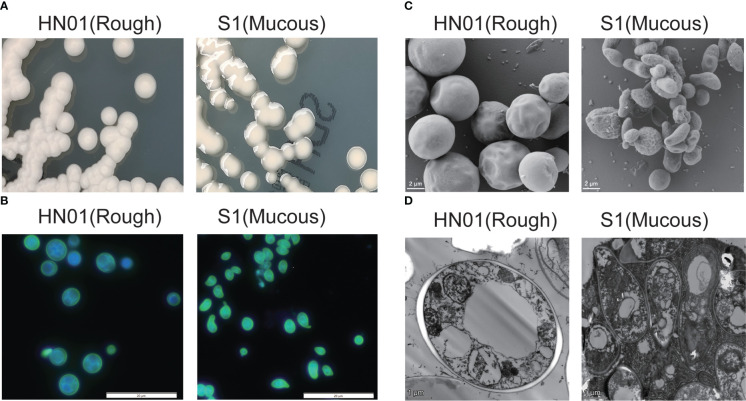
Phenotypic characterization of *P. wickerhamii* HN01 (rough) and S1 (mucous) **(A)** Colonies on Sabouraud dextrose agar for 3 days at 35°C. **(B)** Details of cell morphology, as seen using optical microscopy (fluorescence stain, ×1,000). **(C)** TEM and **(D)** SEM (×6,000).

*P. wickerhamii* HN01 colony morphology was consistent with that of ATCC16529; sporangia and sporangiospores were globose, and the capsule was absent. The diameter of *P. wickerhamii* HN01 sporangia measured 2.69–5.90 μm. After incubation on SDA at 35°C for 3 days, the colonies were creamy white, raised, and circular, with a smooth surface and a diameter of 3–5 mm (**Figure 1A**). *P. wickerhamii* HN01 can grow at 28 or 37°C.

*P. wickerhamii* S1 sporangia were globose to ellipsoidal or angular, sporangiospores were globose to angular and water drop-shaped, and the capsule was absent. The diameter size of *P. wickerhamii* S1 sporangia on average was 1.53-3.65 μm. After incubation on SDA at 35°C for 3 days, colonies were creamy white, glistening, circular, raised, and slimy, with a smooth surface and margins, and were up to 3–5 mm in diameter (**Figure 1A**). *P. wickerhamii* S1 grew well at 28 and 37°C. The cell wall thickness of the HN01 and S1 strains were 0.14 and 0.05 μm, respectively.

### 3.2 Bioinformatics analysis and interpretation of morphological differences between *P. wickerhamii* strains HN01 and S1

**Figure 2:**
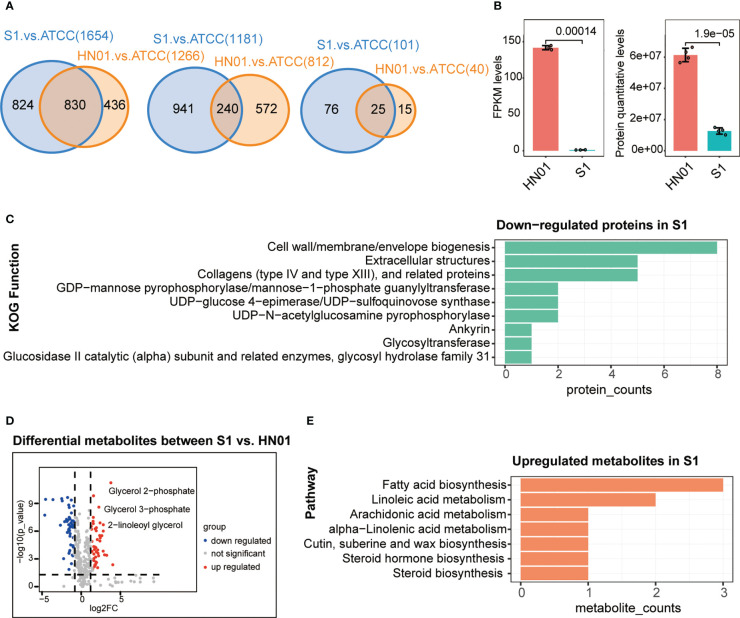
Multi-omics profiles of three *P. wickerhamii* strains ATCC16529, S1 (mucous), and HN01 (rough). **(A)** Venn diagram showing overlap of differentially expressed genes (left), proteins (middle), and metabolites (right) between *P. wickerhamii* strains ATCC and S1/HN01. Numbers indicate the count of differentially expressed genes, proteins, and metabolites. **(B)** Expression levels of mannan endo-1,4-β-mannosidase in *P. wickerhamii* strains HN01 and S1. (Student’s t-test). **(C)** Functional annotation of significantly down-regulated proteins in S1 compared to HN01. **(D)** Differentially expressed metabolites between strains HN01 and S1. **(E)** Enriched pathways of significantly upregulated metabolites in strain S1 compared to HN01.

To better understand the differences at the macro- and micromorphological levels between the two *P. wickerhamii* strains S1 and HN01, we studied their transcriptome, proteome, and metabolome data for comparison. These data were highly reproducible (**Figure S1**). The results showed that the number of DEGs between *P. wickerhamii* strains S1 and ATCC16529 was much higher than that between HN01 and ATCC16529. This was also true for the DEPs and metabolites (**Figure 2A**). This is consistent with previous results showing that strains HN01 and ATCC16529 evolved closely (Guo et al., 2022).

Interestingly, we found that mannan endo-1,4-β-mannosidase was significantly downregulated in S1 compared to that in HN01 (**Figure 2B**). This produced less glucan and mannan, which are the two main components of the algal cell wall. Consequently, this contributed to a thinner cell wall in the mucous strain compared to that in isolate S1 (**Figure 1D**).

Next, we performed a functional annotation of the significantly downregulated proteins in *P. wickerhamii* S1. Strikingly, the results showed that they were enriched in cell wall-related functions such as cell wall/membrane/envelope biogenesis, extracellular structures, collagens (type IV and XIII), and related proteins such as GDP-mannose pyrophosphorylase/mannose-1-phosphate guanylyltransferase (**Figure 2C**). These results are consistent with the transcriptome results, further suggesting that the downregulation of mannose-related enzymes may be responsible for the thin cell wall in S1.

The mucoid appearance of the S1 strain was likely due to the secretion of abnormal metabolites. Therefore, we identified differential metabolites between S1 and HN01, including significantly increased glycerol-related metabolites in S1 such as glycerol 2-phosphate, glycerol 3-phosphate, 2-linoleoyl glycerol, etc. (**Figure 2D**). This may partially explain the mucoid appearance of S1.

We further analysed the pathways in cases in which metabolites were significantly upregulated in S1 compared to HN01. The results showed that they were enriched in glycerol-related pathways, such as linoleic acid metabolism, fatty acid metabolism, and arachidonic acid metabolism (**Figure 2E**). Taken together, we speculate that the mucoid appearance of S1 may be caused by an increase in linoleic acid, glycerol, and other metabolites.

### 3.3 Cytotoxicity of *P. wickerhamii* S1 strain to macrophages is significantly lower than that of HN01

**Figure 3:**
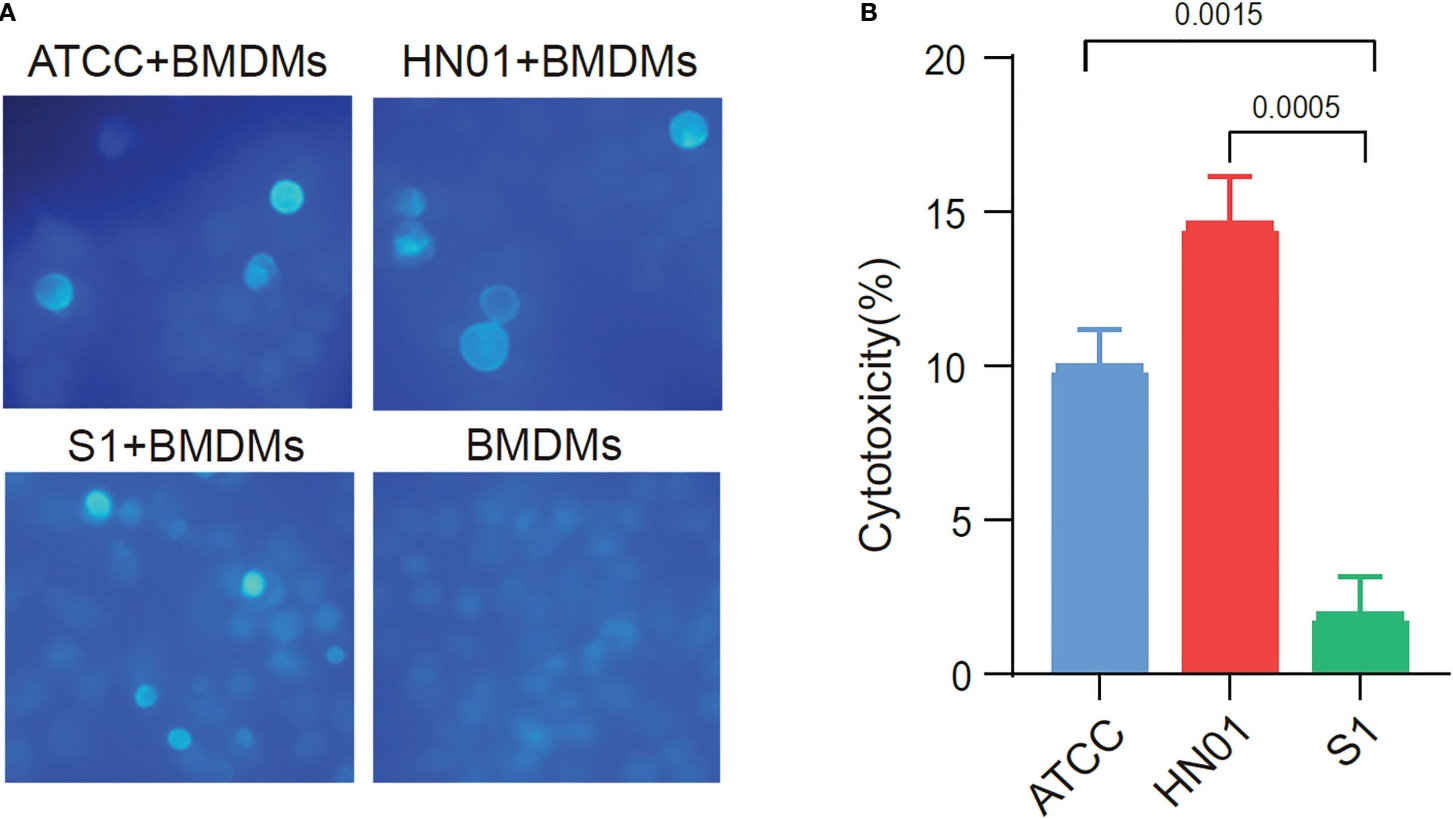
Cytotoxicity of ATCC16529, S1 (mucous) and HN01 (rough) strains to bone marrow-derived macrophages. **(A)** Three *P. wickerhamii* strains and bone marrow-derived macrophages (BMDMs) were co-cultured and stained with calcofluor white, **(B)** Three *P. wickerhamii* strains showed different macrophage cytotoxicity (%).

To compare the differences between the two strains with different morphologies in inducing host immune cell responses, we investigated the interaction of *P. wickerhamii* and macrophages co-cultured *P. wickerhamii* strains with macrophages. RAW264.7 macrophages were co-cultured with *P. wickerhamii* at a ratio of 1:1 (MOI = 1) (**Figure 3A**). Compared to the LDH of *P. wickerhamii* in a medium without macrophages, we found that the macrophage cytotoxicity of isolate HN01 was 14.52% at 24 h, but S1 showed lower viability (1.87%) compared to HN01. However, the cytotoxic effects of *P. wickerhamii* ATCC16529 were 9.91%. These results show that S1 caused much less damage to macrophages than strains HN01 and ATCC16529.

Thus, *P. wickerhamii* S1 showed lower cytotoxicity against macrophages, which could help *P. wickerhamii* escape macrophage clearance. Furthermore, the mechanisms underlying the innate immune response against *P. wickerhamii* should be elucidated in future studies. Taken together, S1 caused less macrophage death than HN01 and ATCC16529 (**Figure 3B**), which may be related to the abnormal thinning of the S1 cell wall.

### 3.4 Strain isolation of *P. wickerhamii* in China based on One Health

**Figure 4:**
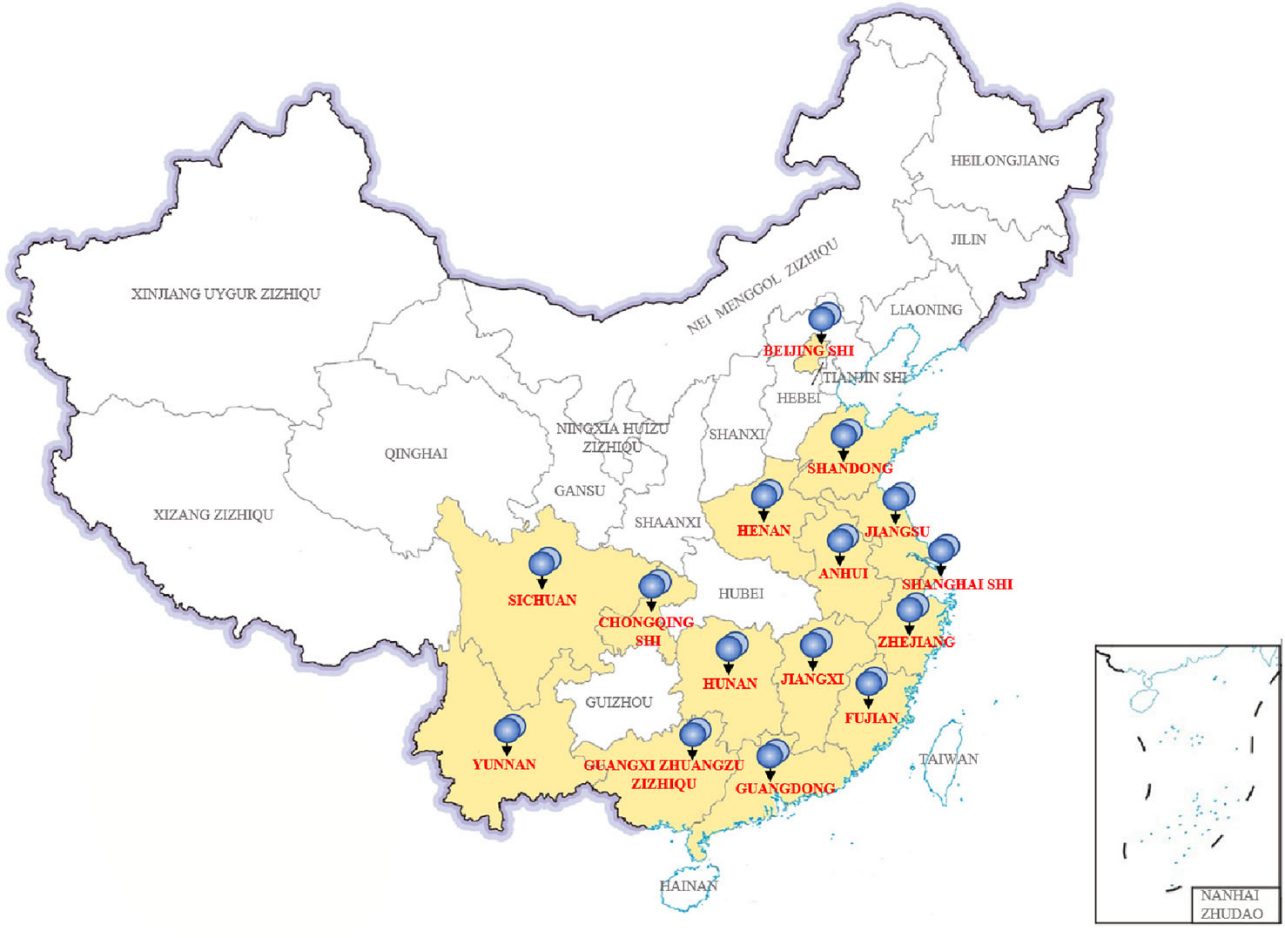
Thirty-one *P. wickerhamii* strains from 15 provinces and cities in China.

In China, we collected 31 *P. wickerhamii* strains that cause human infections, mainly from 15 provinces and cities (**Figure 4**). These cases were mainly concentrated in provinces and cities in southern China with developed waters, including domestic sewage and artificial watercourses. Shanghai, Fujian, Jiangxi, Zhejiang, and Guangxi had the highest separation rates of *P. wickerhamii* strains at 16.13% (5/31), 12.90% (4/31), 12.90% (4/31), 12.90% (4/31), and 9.68% (3/31), respectively. There have also been cases of *P. wickerhamii* bloodstream infections that have led to death, although most cases involved multiple skin infections. In China, owing to the lack of understanding of the pathogenicity and biology of *P. wickerhamii*, the correct diagnosis rate of *P. wickerhamii* infections is very low. For example, no cases of infection were found in the Hubei and Guizhou provinces.

According to the literature, *P. wickerhamii* infections have also been reported in Hong Kong and Taiwan. Unfortunately, there are no studies involving a large-scale investigation of *P. wickerhamii* contamination in both natural and artificial waterbodies in China. However, many reports have shown that *P. wickerhamii* is widespread in the environment and can cause infections in animals. With further research, new species and atypical strains of *Prototheca* may be found in the environment and in animal or human infections.

## 4 Discussion

*Prototheca* are non-photosynthetic, unicellular microalgae associated with severe infections (Nelson et al., 1987). *Prototheca* are the only microalgae which have been reported to infect animals and humans, causing many forms of *Prototheca* infection (Jagielski et al., 2019b). Standard treatment guidelines for protothecosis have not yet been established in humans worldwide (Yamashita et al., 2022). Microalgae often exhibit high levels of resistance to many antimicrobial agents, and there is a low correlation between *in vitro* susceptibility and clinical responses (Lass-Flörl and Mayr, 2007). The *P. wickerhamii* genome encodes several virulence factors, including those identified in *Candida albicans* and *Trichophyton rubrum*. These virulence factors are involved in host invasion and elicitation of the adaptive stress response. The ATPase and malate dehydrogenase genes encode proteins that are potential virulence factors in *Prototheca* (Bakuła et al., 2021).

Culture-based studies can be used to screen for *Prototheca* microalgal isolates from the environment. *P. wickerhamii* has been isolated from food and drinking water; therefore, we need to understand the ecology and pathogenesis of *P. wickerhamii* in different humans, environments, and animals using a One Health approach. With the effects of global climate change, the medium to pathogenic potential of *P. wickerhamii* should be considered from a One Health perspective. The most frequently isolated *Prototheca* species were *P. wickerhamii* (33%), *P. pringsheimii* (23%), and *P. cerasi* (14%) in Poland. Moreover, higher temperatures combined with high humidity would enhance the growth of *P. wickerhamii* in the environment (Jagielski et al., 2022). This study provides an in-depth understanding of the genome sequences of different strains of *P. wickerhamii* isolated from clinical specimens, contributes to a basic understanding of this alga, and explores future prevention and treatment strategies (Guo et al., 2022).

Multiomics analysis and molecular typing of strains isolated from the environment, together with those causing infections, could shed light on the aetiology of *P. wickerhamii* infections. The treatment of human skin and systemic infections by *Prototheca* includes antifungal agents such as azoles. *Prototheca* shares similar features with yeast-like fungi, specifically the presence of ergosterol in the cell membrane, which is approximately three times lower than that in fungal membranes (~4% vs. ~12%). Intravenous amphotericin B is the most effective treatment for *P. wickerhamii* infections. Fluconazole and itraconazole showed cure and improvement rates of 65% and 71%, respectively. Oral itraconazole or fluconazole was recommended for relatively mild cases, and intravenous amphotericin B was used to treat serious infections that azole treatment failed to treat (Todd et al., 2018). *In vitro* tests have confirmed that ravuconazole is more effective than other azoles in treating *P. wickerhamii* infections (Watanabe et al., 2021). The echinocandins are generally ineffective because polysaccharides in the *P. wickerhamii* cell wall are typically β-1, 4-bonded. The development of more effective drugs is required for the treatment of animal and human protothecosis (Kano, 2020). *In vitro* studies have shown that silver nanoparticles are novel algicidal agents (Jagielski et al., 2018).

Studies have shown that MoTup1 is necessary for fungal pathogen virulence. CaTUP1 and Tup1 are related to transcription factors and inhibitors, respectively. Therefore, transcriptional regulation may be important in the pathogenesis of human protothecosis (Guo et al., 2022). Of the 245 proteins identified in *Prototheca zopfii* from the iTRAQ-LC-MS/MS proteomic analysis, only 22 proteins were clearly identified, most of which were putative uncharacterised proteins (Liu et al., 2018). This study identified the reason for the mucoid appearance of the S1 strain, which was more likely due to the mucous secretion of abnormal metabolites, such as glycerol 2-phosphate, glycerol 3-phosphate, and 2-linoleoyl glycerol.

Expanding knowledge based on genome sequencing (Bakuła et al., 2021; Guo et al., 2022) and transcriptomic, proteomic, and metabolomic analyses of *P. wickerhamii* may contribute to a better understanding of the pathways of infection and pathogenesis. The *Prototheca* cell wall is a complex structure composed primarily of β-glucan and mannan. Pathogenic *Prototheca* can expose specific pathogen molecular patterns at their cell surface, which can be recognised by human or animal pattern recognition receptors, potently stimulating the immune response. *Prototheca* should be able to alter the cell wall architecture, enabling them to escape the immune system. Glycoside hydrolases cleave glycosidic bonds between polysaccharides and oligosaccharides and are important virulence factors in many fungal species. ROT2 and SKN1 in *C. albicans* have been linked to cell wall synthesis, and mutants of these genes show decreased *in vitro* virulence (Bakuła et al., 2021). In the present study, we examined the macrophage cytotoxicity of strains S1, HN01, and ATCC16529, which supported the importance of intact cell walls in the induction of immune responses. These results prompted us to closely examine the structural complexity of *P. wickerhamii* cell walls (Wang et al., 2022).

This study has some limitations. *P. wickerhamii* genotypes exhibited growth differences when cultured at different temperatures. Considering the limited data on the pathogenicity of *P. wickerhamii* and that many proteins in the protosheath genome are still annotated as hypothetical proteins, it is reasonable to identify a relatively high proportion of functionally unknown proteins (Liu et al., 2018). The environmental isolate ATCC16529 at 35°C may have induced the expression of stress-related proteins. However, environmental *Prototheca* species have been known to colonise the human skin and other parts or systems, and growth is optimised between 25°C and 37°C. Our study aims to compare the phenotypic characteristics of *P. wickerhamii* clinical strains S1 and HN01. Comparing the differences between these two strains with different morphologies in terms of macrophage cytotoxicity, we found that S1 was significantly less cytotoxic than HN01. The proteomics results were consistent with the transcriptome results, further suggesting that the downregulation of mannose-related enzymes may indeed be responsible for the thin cell wall in S1. Mannan endo-1,4-β-mannosidase may be linked to cell wall synthesis and shows decreased virulence in *P. wickerhamii in vitro*. From a One Health perspective, we will gain a deeper understanding of the ecology, aetiology, pathogenesis, and transmission routes of *P. wickerhamii* between different hosts, environments, and habitats.

## 5 Conclusion

In this study, we show for the first time the phenotypic characterisation of different pathogenic isolates of *P. wickerhamii* clinical strains S1 and HN01. Our data provide evidence of differences in protein expression between these two isolates. The results indicate that S1 differs from HN01 in terms of cell wall thickness, composition, and macrophage cytotoxicity. Further transcriptomic, proteomic, and metabolomic studies will provide valuable information on the pathogenic mechanisms of *P. wickerhamii* infection. Future studies investigating mannan endo-1,4-β-mannosidase and cell wall function-related proteins are needed, such as cell wall/membrane/envelope biogenesis, extracellular structures, collagens (type IV and XIII) and related proteins, and GDP-mannose pyrophosphorylase/mannose-1-phosphate guanylyltransferase.

## Data availability statement

The datasets presented in this study can be found in online repositories. The names of the repository/repositories and accession number(s) can be found in the article/**Supplementary Material**.

## Ethics statement

The studies involving human participants were reviewed and approved by the ethics committee of Shanghai East Hospital, Tongji University School of Medicine (No. 2020-163).

## Author contributions

Conceptualisation: JG, JC and TL. Formal analysis: JG and JC. Funding acquisition: WW and CJ. Methodology: JG, JC, TL, LG, WW and CJ. Supervision: WW and CJ. Writing the original draft: JG and JC. Writing, review, and editing: WW and CJ. All authors contributed to the article and approved the submitted version.
