# Supplementary material for: Integration of transcriptomics, proteomics, and metabolomics data for the detection of the human pathogenic Prototheca wickerhamii from a One Health perspective
Source: Front Cell Infect Microbiol. 2023 May 5;13:1152198. doi: 10.3389/fcimb.2023.1152198 (PMC10196235; doi:10.3389/fcimb.2023.1152198)
Supplement: Supplementary file 1 [file DataSheet_1.pdf]

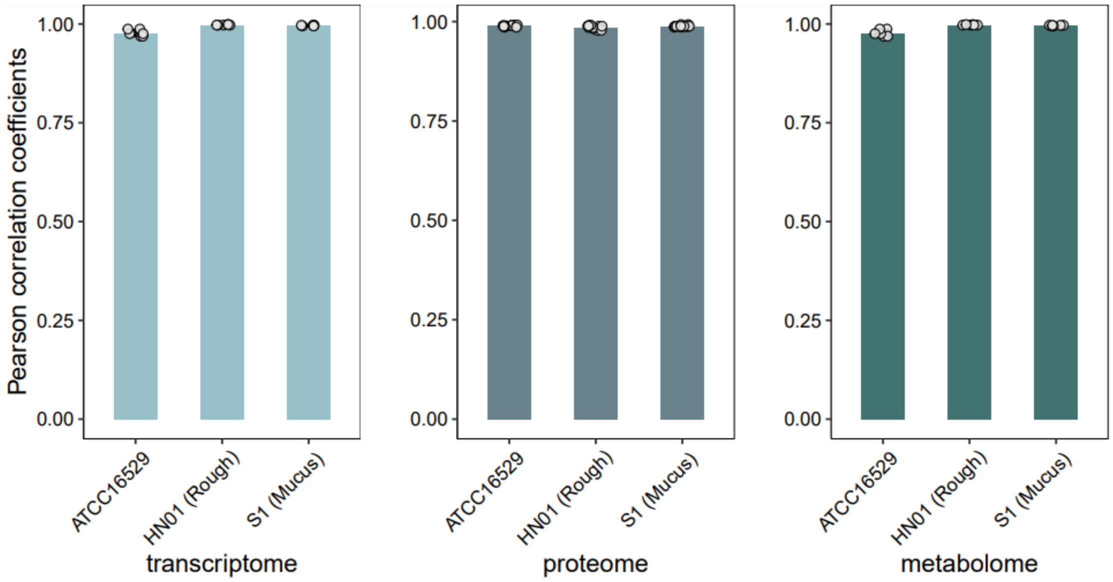

FIGURE S1. Pearson correlation coefficients for transcriptome (left), proteome (middle), and metabolome (right) data from biological replicates of three *P. wickerhamii* strains ATCC16529, HN01 (rough), and S1 (mucous) (transcriptome, each 3 replicates; proteome, each 4 replicates; metabolome, each 6 replicates).
